# Prenatal smoking and child psychopathology associations by age and sex in the ECHO cohort

**DOI:** 10.1017/S095457942610128X

**Published:** 2026-04-06

**Authors:** Kristine Marceau, Andrew Law, Karen J. Derefinko, Olivia C. Robertson, Sara S. Nozadi, Lijun Li, Chang Liu, Leslie D. Leve, Jody M. Ganiban, Nicole Bush, Cathi Propper, Briana Moore, Amy J. Elliott, Catrina A. Calub, Theresa M. Bastain, Donghai Liang, Julie B. Schweitzer, Ban Al-Sahab, Rebecca J. Schmidt, Heather E. Volk, Jenae M. Neiderhiser

**Affiliations:** 1 Human Development and Family Science, College of Health and Human Sciences, Purdue University, USA; 2 Johns Hopkins University Bloomberg School of Public Health, USA; 3 Department of Preventive Medic, The University of Tennessee Health Science Center, USA; 4 USDA/ARS Children’s Nutrition Research Center, Department of Pediatrics, Baylor College of Medicine, Houston, USA; 5 The University of New Mexico Health Sciences Center, USA; 6 Department of Psychology, The Pennsylvania State University, USA; 7 Department of Psychology, Washington State University, USA; 8 Prevention Science Instistute, University of Oregon, USA; 9 Psychology, George Washington University, USA; 10 Departments of Psychiatry and Pediatrics, University of California, San Francisco, USA; 11 School of Nursing, The University of North Carolina at Chapel Hill, USA; 12 Department of Epidemiology, Colorado School of Public Health, USA; 13 Avera Research Institute, USA; 14 Pediatrics, University of South Dakota Sanford School of Medicine, USA; 15 Department of Psychiatry and Behavioral Sciences; MIND Instistute, University of California, Davis, USA; 16 School of Public Health, Washington University in St. Louis. USA; 17 Gangarosa Department of Environmental Health, Emory University Rollins School of Public Health, USA; 18 Department of Psychiatry and Behavioral Sciences and MIND Institute, University of California, Davis, USA; 19 Department of Family Medicine, Michigan State University, Department of Family Medicine, USA; 20 Department of Public Health Sciences, MIND Institute, School of Medicine, University of California, Davis, USA; 21 Department of Mental Health, Johns Hopkins University Bloomberg School of Public Health, USA

**Keywords:** Smoking during pregnancy, Environmental Influences on Child Health Outcomes (ECHO), internalizing, externalizing, severity and directionality

## Abstract

Maternal tobacco smoking during pregnancy (MSDP) is associated with an increased risk of child externalizing problems. It remains unclear whether these associations are externalizing-specific, or better explained by comorbidity between externalizing and internalizing domains, or vary by age and sex. To address comorbidity and differentiation between domains, we leveraged the severity-directional model of psychopathology. Severity reflects the overall level of psychopathology symptoms across both domains (high levels of severity can only be reached by having symptoms of both types simultaneously), whereas directionality captures the balance/differentiation of internalizing vs. externalizing symptoms regardless of number of total symptoms. Participants included 16,335 children aged 1–19 years old (47.78% female, 58.17% White, 75.46% non-Hispanic) from 55 U.S.-based cohorts within the Environmental Influences on Child Health Outcomes (ECHO) consortium. MSDP predicted differentiation toward externalizing problems in 2-year age bins 1–2 through 7–8 and 13–14 years; remaining (non-significant) age bins had similar magnitudes. MSDP predicted higher symptom severity in all age bins. Findings likely reflect a combination of MSDP associations with comorbid symptom severity and specificity toward externalizing problems, with little evidence of age or sex differences. Additional analyses explored e-cigarette use, other prenatal substance use, and postnatal smoke exposure; associations were sparse and unsystematic.

## Introduction

Maternal tobacco smoking during pregnancy (MSDP) has emerged as a key risk factor for developing externalizing problems during childhood and adolescence (He et al., 2020; Ruisch et al., 2018). However, drawing on principles of multifinality (Cicchetti, 2016) and evidence of high comorbidity of externalizing with internalizing problems during childhood and adolescence (Willner et al., 2016), MSDP may be a general risk for psychopathology or a specific risk for externalizing problems. Reflecting a long-held conceptualization of disorders as distinct syndromes in psychology and psychiatry, previous studies have largely not examined whether MSDP creates a general risk for psychopathology or whether it influences the type of psychopathology (i.e., externalizing vs. internalizing disorders). The present study addresses this gap by leveraging the severity-directionality model to investigate associations between MSDP with comorbid vs. externalizing-specific psychopathology symptoms. In this model, symptom severity refers to the overall level of psychopathology symptoms across both domains and reflects comorbidity because high levels of severity can only be reached by having symptoms of both types simultaneously. Symptom directionality is an orthogonal score that captures the balance or differentiation of internalizing vs. externalizing symptoms regardless of number of total symptoms. This model (explained in more detail below) thus allows for direct examination of the comorbidity and specificity of symptoms in the internalizing and externalizing domains.

In addition, there have been few evaluations of whether the effects of MSDP vary as a function of child age and sex. Studies typically examine a single age or developmental period (e.g., adolescence), but evidence suggests that MSDP is related to at least externalizing difficulties in childhood and adolescence, and associations may even persist into adulthood (Tien et al., 2020). There is evidence of this for boys and girls (Xie & Mao, 2024) with some (mixed) evidence that effects may be stronger for males (e.g., Hutchinson et al., 2010; D‘Onofrio et al., 2010; Fergusson et al., 1998). We therefore add to the literature by leveraging a large sample capable of assessing sex differences in MSDP-psychopathology symptom associations in 2-year age bands spanning toddlerhood through late adolescence.

### MSDP and psychopathology symptoms

Meta-analytic evidence shows clear associations of MSDP with externalizing problems in childhood and adolescence (He et al., 2020; Ruisch et al., 2018), although genetic and familial environmental influences shared by mothers and youth often attenuate these associations (Haan et al., 2022; Havdahl et al., 2022; Marceau et al., 2018; Marceau et al., 2019). Fewer studies have examined MSDP and child internalizing problems; a meta-analysis identified significant small associations (Duko et al., 2020), although findings for internalizing problems are sparse and generally weak or null (Tien et al., 2020), especially in studies that control for familial (genetic and rearing environmental) confounders (Bonello et al., 2023; Ekblad et al., 2020).

#### Severity and directionality

One of the challenges of examining whether MSDP risk is externalizing-specific vs. general and/or contributing to risk for comorbidity is the high correlation between internalizing and externalizing domains, particularly in adolescent samples (Marceau et al., 2022). One solution is to reorganize psychopathology symptoms into two orthogonal scores (Essex et al., 2003; Marceau et al., 2022). As a heuristic example, assuming that there are 10 symptoms for externalizing and 10 symptoms for internalizing problems that can be endorsed, the sum of the two scores would equal symptom severity, and the difference would equal directionality (in this example, we will assume internalizing–externalizing, where more positive values indicate more internalizing, whereas more negative values indicate more externalizing symptoms). An individual with 10 internalizing symptoms and 10 externalizing symptoms would have the maximal value for severity (20), but a directionality score of 0 (indicating perfect balance 10–10). A directionality score of 0 could also be achieved by having 5 of each type of symptom or no symptoms at all, making it orthogonal to the severity score. An individual with 10 internalizing symptoms and 0 externalizing symptoms would have a moderate severity score (10), but a directionality score indicating a strong preponderance of internalizing symptoms (10). Moderate severity scores (e.g., 10) could be achieved by high numbers of a single type of symptom (e.g., all 10 internalizing symptoms), but more often is achieved by some type of balance (e.g., 3 internalizing and 7 externalizing symptoms). This latter example would yield a directionality score of 3 – 7 = −4 indicating a moderate preponderance of externalizing symptoms. In practice, many measures do not have equal numbers of items in each domain nor yes/no response options, and so the scale scores for internalizing and externalizing problems are entered into principal components analyses extracting exactly two factors to achieve the same conceptual scores (see Essex et al., 2003; methods section).

The first score, *symptom severity*, is conceptually similar to a second-order “*p*-factor” in hierarchical models of psychopathology (Caspi & Moffitt, 2018) and indexes comorbidity, with youth with comorbid problems demonstrating the highest scores (Marceau et al., 2022). The second score, *symptom directionality*, indexes differentiation of problem type on a continuum from purer internalizing problems (i.e., highly positive values) to purer externalizing problems (i.e., highly negative values); this measure is not captured by other models of psychopathology (Marceau et al., 2022). The use of symptom directionality in the current study is novel and informative because most studies examine internalizing and externalizing problems separately to avoid common multicollinearity issues due to their comorbidity, preventing conclusions about whether effects are driven by comorbid symptoms or whether MSDP affects symptom differentiation. Further, leveraging symptom severity and directionality scores can help to strengthen inferences about multifinality in associations of MSDP and psychopathology symptom outcomes. If associations are found between MSDP and internalizing, externalizing, and severity scores, this is strong evidence that multifinality is a key concept for understanding the effects of MSDP on development. If, in contrast, associations are strongest for differentiation toward externalizing, regardless of severity of symptoms, then this evidence would refute the concept of multifinality for the role of MSDP, instead pointing to specialized pathways that increase risk of specifically externalizing problems even in the context of comorbidity observed in childhood and adolescence.

#### Associations with severity and directionality

Thus far, the only study to examine MSDP in relation to severity and directionality of youth internalizing and externalizing psychopathology did so in a sibling comparison study of 8 to 15-year-old youth, a design that controls for some genetic and familial environmental confounding (Ekblad et al., 2020). They found that siblings exposed to more MSDP were more likely to show differentiation towards externalizing problems than their co-sibling (a MSDP-directionality association), but no association between MSDP and symptom severity within families.

Other studies have examined broader indices of perinatal risk that include MSDP. Total obstetric complications (including MSDP) was associated with differentiation toward externalizing problems relative to internalizing problems at age 4.5 years in the context of low marital hostility and low genetic risk for substance use (Neiderhiser et al., 2016). A large longitudinal study found that prenatal substance use exposure predicted externalizing directionality at age 13, but only for boys (Marceau et al., 2021). However, bivariate associations of prenatal substance use with severity for boys and girls did not survive adjustment for familial confounds (Marceau et al., 2021), consistent with prior findings (Ekblad et al., 2020). In all, the literature to date suggests that MSDP is a specific risk factor for externalizing problems and only associated with internalizing problems through comorbidity.

### Age and sex differences

The literature to date is not systematic with regard to testing sex and age differences in MSDP-psychopathology symptom associations, though we might expect them based on (1) sex differences in developmental trajectories of internalizing and externalizing across childhood and adolescence and (2) fetal programming hypotheses that posit sex-specific programming effects and findings suggesting MSDP to be particularly problematic for boys, as described below.

#### Developmental trajectories of internalizing and externalizing

There are age and sex differences in internalizing and externalizing symptoms across childhood and adolescence, with externalizing problems more common earlier in development and among boys, but internalizing problems more often showing adolescent onset and more common among girls (Zahn-Waxler et al., 2008). However, these changes are not uniform. For example, in a study spanning ages 4 to 16 years, internalizing symptoms decreased across early childhood (age 4–8), increased slightly in late childhood (age 8–10), remained stable through about age 14 and increased between 14 and 16 years (Katsantonis, 2024). In contrast, externalizing behavior typically decreases from early childhood though preadolescence but increases during adolescence (Petersen et al., 2015). From ages 3 to 12, conduct problems (e.g., externalizing) decreased whereas emotional symptoms (e.g., internalizing) increased for boys and girls (Álvarez-Voces et al., 2024). Notably, the decrease in conduct problems was slightly quadratic (accelerating) for boys but not girls, hinting at sex differences in trajectories, though there were not sex differences for emotional problems. Together, the developmental trajectories of both internalizing and externalizing problems are nonlinear across early childhood through adolescence.

#### Severity and directionality

Conceptually, severity and directionality may change developmentally as the levels and balance of internalizing and externalizing problems shift over time. However, there has not been systematic work examining developmental changes in severity and directionality. The variance explained by severity and factor loadings for severity and directionality in a sample of adopted children were similar at age 11 (81%, severity factor loadings = .91, directionality = ± .42; Marceau et al., 2024) compared to at age 4.5 (83%, severity factor loadings = .90, directionality = ± .44; Neiderhiser et al., 2016). In a study of 11- to 15-year-old adolescents at a first assessment and followed up approximately two years later, Pearson’s correlations of symptom severity and directionality scores were *r* = .74 and *r* = .56, respectively (Marceau et al., 2015). Together, this literature provides hints that severity and directionality scores may operate similarly within age bands and samples and that there is moderate-to-high stability over time, although much more work is needed to understand the development of psychopathology in this conceptual framework. The present study will contribute to our understanding of how severity and directionality operates across time (e.g., similarly within age bands and samples) by constructing severity and directionality within age bins, although it cannot speak to developmental patterns due to its cross-sectional nature.

#### Sex-specific programming effects of MSDP

Mounting evidence suggests that male fetuses are more at risk for negative growth and development outcomes stemming from in utero exposures (Sutherland & Brunwasser, 2018). Several studies have shown that maternal smoking during pregnancy may have sex-specific fetal programming effects. For example, MSDP associations with externalizing symptoms were stronger for males than females during early childhood (Hutchinson et al., 2010), middle childhood (Wakschlag & Hans, 2002; D‘Onofrio et al., 2010), adolescence (Fergusson et al., 1998), and MSDP associations with criminal arrest histories and substance abuse in adults were also stronger for males than females (Brennan et al., 2002).

#### Sex and age differences in MSDP-psychopathology associations

There is some evidence that MSDP is associated with within-person increases in externalizing (but mixed evidence for internalizing) problems over childhood and adolescence (Ashford et al., 2008; Sutin et al., 2017). This is important evidence of the role of MSDP in the development of psychopathology, but trajectory-based analysis does not allow for identification of peak ages at which MSDP effects are strongest. Instead, examining associations at multiple ages can help to identify potentially sensitive developmental periods for the association of MSDP and psychopathology. A recent study found evidence of potentially causal associations of perinatal maternal smoking on ADHD diagnosis in both boys and girls during multiple developmental periods by examining separate diagnostic categories: childhood-onset, late-onset, and persistent diagnostic categories (Xie & Mao, 2024). This study demonstrates that there may not be developmentally sensitive periods of MSDP on psychopathology, although it is limited for inferring developmentally sensitive periods of MSDP associations by examining only three large age-related categories (childhood, late, both) and by examining only ADHD outcomes. In studies examining comorbid symptom severity and directionality, Marceau et al. (2021) and Neiderhiser et al. (2016) focused on two very different age ranges. Further, the associations reported were in the context of complex models which precluded the explicit test of sex differences in Neiderhiser et al. (2016). The sample used by Ekblad and colleagues (2020) included 173 families with a broad age range and was unable to examine whether there are key developmental periods driving the associations they found or if the associations are uniform across middle childhood through mid-adolescence. The sample size and sibling gender composition precluded tests of sex differences in that study as well.

Taken together, there are clear gaps in the literature understanding whether there are sex and age differences in MSDP-psychopathology symptom associations. This information is critically needed to identify whether there are sensitive periods for boys and girls for the emergence of externalizing (or comorbid) problems related to MSDP exposure. If present, sensitive periods may indicate prime developmental periods to intervene with children to prevent externalizing problems in MSDP-exposed youth. In contrast, the absence of sensitive periods would point to the importance of preventing MSDP exposure and focusing on interventions to reduce externalizing problems across childhood and adolescence.

### Present study

We leveraged harmonized data from the Environmental Influences on Child Health Outcomes (ECHO) Cohort, which provides a considerably larger sample size than prior studies and includes participants across childhood and adolescence (1–18 years old). Specifically, we constructed severity and directionality scores within two-year age bins (e.g., using data from 1–2-year-olds, 3–4-year-olds, etc. as stratified samples). We tested associations in each two-year age bin to examine patterning in associations by age. We also tested for sex differences within each age bin. We controlled for demographic covariates and, additionally, fit models controlling for prenatal use of drugs other than tobacco (i.e., opioids, cannabis, alcohol, and other illicit drugs) and postnatal secondhand smoke exposure to isolate the MSDP effect.

Based on prior studies of symptom severity and directionality, we hypothesized that (1) MSDP would be related to symptom directionality (specifically, differentiation toward externalizing vs. internalizing symptoms), and (2) MSDP would also be related to symptom severity regardless of problem type (indexing comorbidity). Age and sex differences are exploratory, as existing evidence was not strong enough to form specific hypotheses, though evidence may point to consistent results across age periods and stronger effects for males than females.

In sensitivity analyses, we compared findings to associations with broadband internalizing and externalizing scores that did not account for comorbidity. In a subset with available data, we examined e-cigarette use as the exposure variable to infer whether cigarette smoke or nicotine exposure would drive findings.

## Methods

### Study population

Data were drawn from the collaborative ECHO consortium (Knapp et al., 2023). The ECHO Cohort consists of 69 cohort study sites across the United States and aims to investigate the effects of early life exposures on child health and development using a common data collection protocol. The ECHO Cohort eligibility criteria and study recruitment have been described previously (Knapp et al., 2023). Local cohort-specific and/or the ECHO single Institutional Review Boards approved the study protocol. Written informed consent and child assent as appropriate was obtained for each participant. We followed the Strengthening the Reporting of Observational Studies in Epidemiology (STROBE) guidelines.

**Figure 1.**
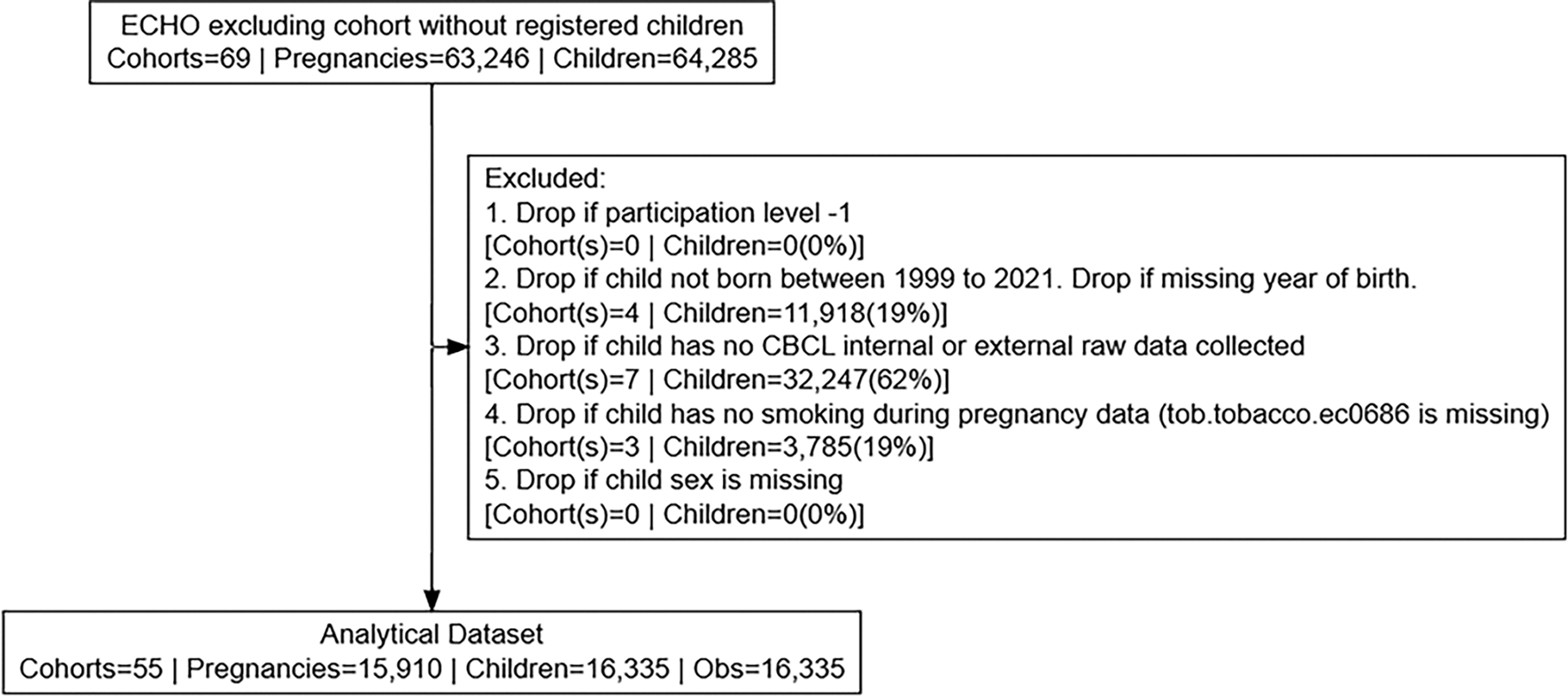
Exclusion flow chart.

Our analytic sample included 16,335 children aged 1–18 years old from 55 cohort sites with harmonized data pertaining to psychopathology symptoms and MSDP. The decision logic for inclusion/exclusion of cohorts and participants is provided in Figure 1. Table 1 shows that the analytic sample (31% of the full ECHO sample) was similar in composition to the full ECHO Cohort on key study variables and demographic characteristics.

### Measures

All measures described below were harmonized by ECHO working groups. Reports and details are available from the ECHO Data Analysis Center.

#### Prenatal smoking or tobacco use

All cohort sites collected prenatal maternal tobacco use, which overwhelmingly reflected maternal retrospective recall of cigarette smoking. Three cohorts reported “prenatal maternal smoking” that reflected tobacco smoking for those samples. We used a binary (1 = yes, 0 = no) indicator of any maternal smoking reported during any part of the pregnancy. Quantity and frequency measures were too sparse across cohorts to include in the present analysis. For some sites, any e-cigarette use during pregnancy (1 = yes, 0 = no) was also available.

#### Psychopathology

Child broadband internalizing problems (including anxiety/depressed problems, withdrawn, somatic complaints, emotionally reactive) and externalizing problems (including attention problems, delinquent, aggressive behaviors) were measured by parent report on the Child Behavior Checklist (CBCL) and/or the Strengths and Difficulties Questionnaire (SDQ).

#### Harmonization

Harmonization of the CBCL and SDQ were conducted by a collaborative ECHO working group and published in (Mansolf et al., 2022), which includes cross-walk tables, IRT, and equipercentile linking methods to ensure the best possible harmonization of these data. We follow the recommendations put forth in that manuscript. Cohorts provided CBCL and/or SDQ data on one of several forms: CBCL-preschool (age 1.5 to 5 y), CBCL-school (age 6–18), and SDQ-2, -4, or -11. Most, but not all cohorts provided item-level data. Harmonization steps included the following steps: (1) Verifying age range: CBCL-preschool records where the child age of assessment was <12m or >72m were set to missing. CBCL-school records where the child age of assessment was <5y12m or >19y were set to missing. (2) Verifying values: Values outside of the published ranges for scale scores were set to missing. (3) Missing data: The percentage of missing items (when available) was small; if <10% of items were missing, the missing item(s) were mode-imputed; when >10% of items were missing, the scale score was set to missing. Only one reporter (prioritizing the childrearing parent) and one assessment per 1-year age bin (prioritizing CBCL) were included in analyses. When only SDQ was available, we used the previously published ECHO consortium conversion of SDQ scores to CBCL scores (Mansolf et al., 2022).

#### Measurement invariance testing

Mansolf and colleagues (2022) also conducted measurement invariance tests across the age 2, 4, and 11 versions of the SDQ and concluded that the SDQ is essentially invariant across its forms. Because we examine age bins for psychopathology, we conducted an item-level analysis with the intention of conducting measurement invariance testing for the CBCL. Not all cohorts provided item-level data to the ECHO consortium. Among those that did, there were some items that had no variance within an age bin. We examined 1y, 2y, 3y, and 5y age bins, but even in the 5y age bins (e.g., age 1–5, 6–10 years etc.), several items (e.g., items 2, 99, and 105 which are all about substance use) showed no variance in that no one endorsed the items for some age bins (item variance analyses are available upon author request). This makes sense, as the substance use items are less developmentally relevant indicators of externalizing problems at 6–10 years compared to during adolescence. Further, some items differ across forms (e.g., those items are not asked in the preschool form for developmentally appropriate reasons) yet are included in the school form because of their developmental appropriateness later. Measurement invariance models cannot be fit with no item-level variance because the first stage of measurement invariance testing, structural invariance, is not met. This supports the rationale for harmonizing measures at the scale level. There were 179 zero-variance items across 1y age bins, but only 44 across 2y age bins. Thus, to balance our desire for a fine-grained analysis with regard to age with the sample size within each age bin and need to reduce structural invariance due to zero-variance items across age bins, we elected to examine 2y age bins. Therefore, we present findings from age bins and separately by gender but encourage readers to consider the pattern of findings as a whole rather than draw direct comparisons about differences across age bins (Putnick & Bornstein, 2016).

#### Severity and directionality

Internalizing and externalizing scores were moderately correlated across age bins (*r* = .49–.75), with no discernible developmental pattern.

Within each age bin, the harmonized CBCL/SDQ summary internalizing and externalizing scores were entered into a Principal Components Analysis (PCA) extracting exactly two scores (using all observed, not imputed, data within each age bracket). The first extracted factor is symptom severity, which explained 74–87% of the variance (eigenvalues within each age bin = 1.49–1.75, factor loadings = 0.88–0.91). Higher scores indicate more symptom severity regardless of type, with the highest levels indicating comorbid problems (Marceau et al., 2022). The second factor is symptom directionality, which explained the remaining 13–26% of variance (with externalizing symptoms loading 0.35–0.51 and internalizing symptoms loading equally but negatively across age bins). Higher positive scores on directionality indicate purer internalizing symptoms whereas more negative scores indicate more pure externalizing symptoms.

#### Covariates

***Cohort site*** was included as a covariate to control for measurement and assessment differences across studies in the consortia, and the ***SDQ/CBCL*** measure indicator was included as a covariate for any age bins in which the measures could vary (e.g., there were no SDQ measures beyond age 11 so it was not included as a covariate in age bins beginning with age 11). A series of demographic variables were controlled: ***Maternal educational status*** was categorized into three categories of the highest level of educational attainment: less than high school or a high school graduate and some college with no degree or an associate’s degree and a bachelor’s degree and above (reference = less than high school). ***Maternal age*** was the age of the biological mother at the birth of the child (centered at 30 years). ***Psychiatric disorder in child*’*s first-degree biological relatives*** was indicated if any of the child’s first-degree relatives (defined as mother, father, sister, brother) ever had a psychiatric disorder (1 = yes, reference = no) assessed at the last/current assessment.

We also controlled for a series of variables to more clearly specify maternal prenatal smoking exposure. These included postnatal secondhand smoke exposure (maternal report of any smoking after the birth of the child) and other substance use during pregnancy (any opioid, cannabis, alcohol, or other illicit drug use).

***Child sex*** was the designated sex at birth (male, female, reference = male) and was included as a covariate in models not explicitly testing for sex differences—it was included as a moderator in models testing sex differences (see below).

### Analytic strategy

All analyses were conducted using R version 3.6.3.

#### Missing data

Missing data were addressed via multiple imputation by chained equation (MICE) performed using the “mice” package; we combined 20 imputed data sets using the “pool” function (Van Buuren & Groothuis-Oudshoorn, 2011). The imputation model was used to impute predictor and covariate data for individuals for whom outcome data were available. Specifically, we conducted a multilevel imputation model including the outcomes (internalizing, externalizing, severity, and directionality) as random effects; the exposure (smoking during pregnancy) and covariates, as well as maternal race, ethnicity, and an identifier of cohort as a random effect; and a participant identifier as a fixed effect (Van Buuren et al., 2006). Missing predictor/covariate variables were imputed; however, no outcome data was imputed since the vast majority of missing data on the outcome were because the measures were not asked in entire cohorts—the level of missing outcome data would be too high for reliably imputed values. Predictive mean matching was used to predict missing continuous values, e.g., maternal age at delivery. Logistic regression was used to impute missing binary values like child sex. Polytomous logistic regression was used to impute missing unordered categorical values, e.g., child race, and the proportional odds model was used to impute missing ordered categorical values, e.g., maternal education (Van Buuren, 2007).

#### Hypothesis testing

Generalized estimating equations (GEE) were used to account for the nesting of individuals within study cohort (e.g., cohort was the cluster variable in all models), run on the 20 imputed datasets. GEE analyses were implemented with a Gaussian distribution, robust standard errors, and an exchangeable correlation matrix implemented using geeglm function from the “geekpack” package (Højsgaard et al., 2006; Yan, 2002; Yan & Fine, 2004). All models were conducted within each age bin. Unadjusted and adjusted GEE were performed to calculate a pooled population means, and pooled standard errors were used to construct the 95% confidence intervals (CI).

The GEE were conducted in a series of steps. First, we conducted unadjusted models. Next, the base covariate-adjusted model included child race and ethnicity, maternal age at childbirth, maternal education, and family history of psychiatric disorder, and sex. Third, sex was included as a moderator. Next, we ran an additional three models to test the specificity and isolate the MSDP association. For these three models, we restricted the sample to participants who had data on the key covariates: (1) we added other substance use during pregnancy to the adjusted model, (2) we added postnatal smoke exposure to the adjusted model, and finally (3) we added both together to the adjusted model. Cohort was a cluster variable to account for nesting of children within cohorts. A 2-sided *p* < .05 after using the Benjamini–Hochberg False Discovery Rate correction was taken as evidence for a statistically significant association.

#### Sensitivity analyses

To aid in interpretation of findings, we examined internalizing and externalizing raw scores and computed standardized estimates to enable comparison across scales^1^. We also examined prenatal e-cigarette use as the focal predictor of severity and directionality in a reduced sample. For completeness, we present the associations between covariates (other prenatal substance use and postnatal secondhand smoke exposure) with symptom severity and directionality after accounting for MSDP.

## Results

Included children (*N* = 16,335) were split fairly equally by sex (47.78% female), and most were White (58.17%), with an additional 2.58% of children identifying as American Indian or Alaska Native or Native Hawaiian or Pacific Islander, 4.63% Asian, 17.79% Black, 11.28% more than 1 race, and 5.26% other race and 24.54% Hispanic (Table 1). MSDP was endorsed for 10.17% of the sample. E-cigarette use was endorsed by 3.90% of the subsample with available data. See Table 2 for prevalence of other prenatal substance use and postnatal secondhand smoke exposure by MSDP status. Additional results are accessible in Supplemental files located at https://osf.io/g8rcb/.

### MSDP and symptom severity

**Figure 2.**
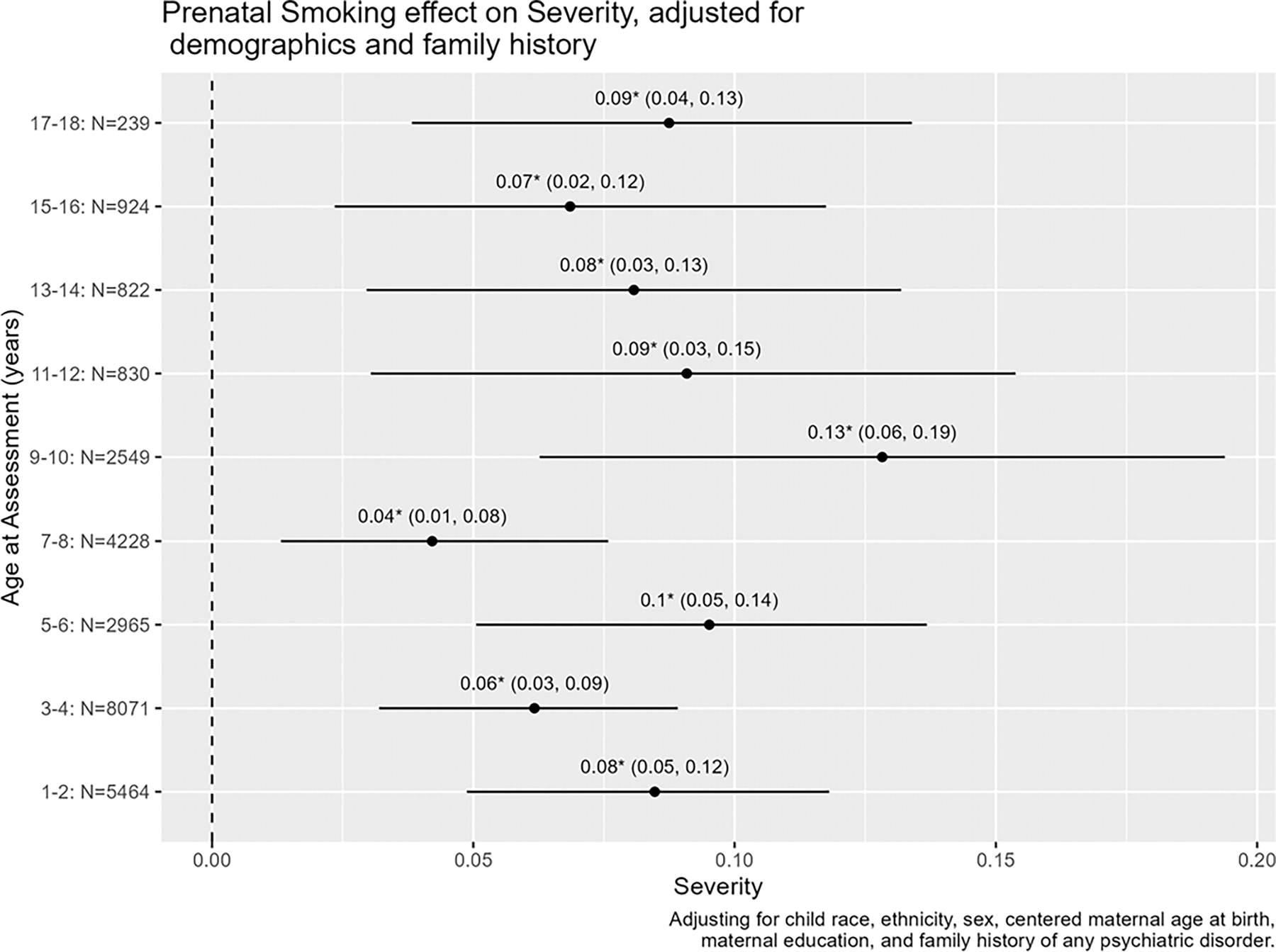
MSDP – Severity associations in adjusted models. Unstandardized point estimates and 95% confidence intervals presented within each age bin. For severity scores, higher positive scores indicate more total, comorbid symptom severity. Estimates are adjusted for sex, centered maternal age at birth, maternal education, and family history of any psychiatric disorder.

In unadjusted models (Supplemental file 1, Section 1.1), MSDP predicted greater symptom severity after FDR correction in all age bins: unstandardized effects ranged from .28, 95% confidence interval: [0.13, 0.52], for 7–8 year olds to .59, 95% confidence interval: [0.33, 0.85], for 9–10 year olds, with estimates and confidence intervals highly overlapping across age bins. Adjusting for covariates (Figure 2), effects only slightly attenuated, and all remained significant after multiple testing. Unstandardized effects ranged from .15, 95% confidence interval: [0.05, 0.27], for 7–8-year-olds to .45, 95% confidence interval: [0.22, 0.68], for 9–10-year-olds, with estimates and confidence intervals again highly overlapping across age bins. In sensitivity tests shown in Supplemental file 1, Section 1.4, the effect completely attenuated (confidence interval included zero) for the later age bins (e.g., 13–14 through 17–18) when any prenatal substance was included as an additional covariate and for age 7–8 and 13–14 when postnatal smoke exposure was included. The effects never completely attenuated for the childhood age bins (1–2 through 5–6) or age bins capturing the early phases of puberty for most youth (9–10, 11–12). Only one out of nine tests showed a statistically significant interaction for sex (Supplemental file 1, Section 1.3): the MSDP association with severity was stronger for boys than girls at age 13–14.

### MSDP and symptom directionality

**Figure 3.**
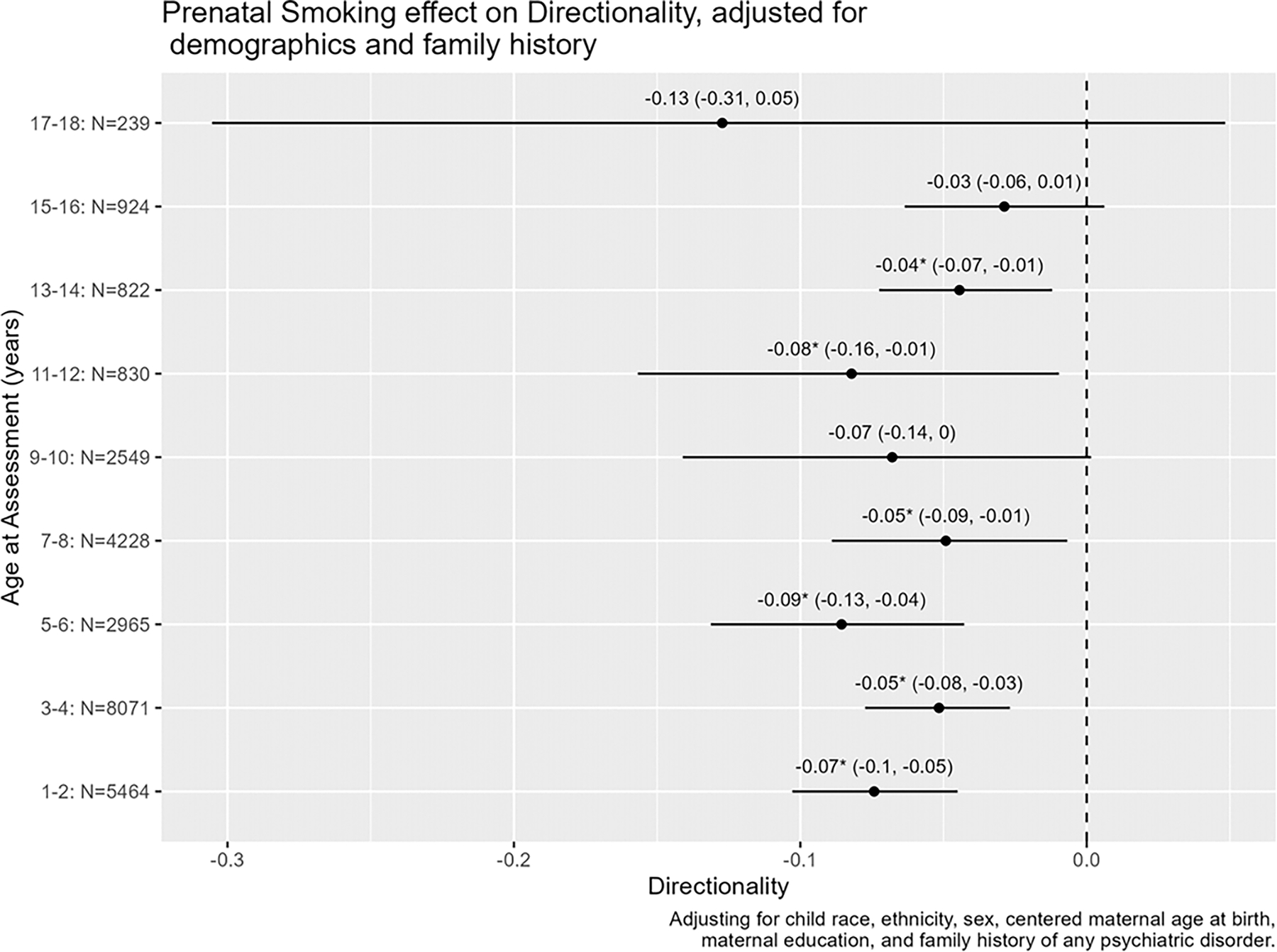
MSDP – Symptom directionality associations in adjusted models. Unstandardized point estimates and 95% confidence intervals presented within each age bin. For directionality scores, higher positive scores indicate differentiation toward purer internalizing problems, 0 indicates balanced internalizing and externalizing symptoms, and more negative scores indicate differentiation toward purer externalizing problems. Estimates are adjusted for sex, centered maternal age at birth, maternal education, and family history of any psychiatric disorder.

In unadjusted models (Supplemental file 1, Section 1.1), MSDP typically predicted differentiation toward externalizing problems: in the earlier age bins, unstandardized *β* ranged from −.09, 95% confidence interval: [−0.16, −0.02] at age 7–8 years to −.14 [−0.22, −0.07] at age 5–6. Exceptions included age 15–16 and 17–18 which had nonsignificant point estimates with confidence intervals including zero (0.01 [−0.03, 0.06]; −0.24 [−.48, .01], respectively). Adjusting for covariates (Figure 3) did not appreciably change associations (Supplemental file 1, Section 1.4), except that the effect for the 9–10y age bin became non-significant (with the same strength of point estimate). In sensitivity tests shown in Supplemental file 1, Section 1.4, the effect completely attenuated (confidence interval included zero) for the 13–14y age bin when any prenatal substance was included as an additional covariate (note that in this model the 9–10y age bin was once again significant). When postnatal smoke exposure was included the age 7–8 and 9–10y age bins showed attenuated effects (of similar magnitude). When controlling for both prenatal and postnatal exposure the association of MSDP with externalizing directionality attenuated in all age bins that converged (e.g., the sample size was too small in the older age bins and the models did not converge). Only one out of nine tests showed a statistically significant interaction for sex (Supplemental file 1, Section 1.3): the MSDP association with a preponderance of externalizing problems specifically was stronger for boys at age 13–14.

#### Sensitivity analyses

##### Internalizing and externalizing

MSDP predicted higher externalizing problems in all age bins, and associations attenuated only minimally in adjusted models (Supplemental file 1, Sections 1.1 and 1.2). MSDP associations with higher internalizing problems were smaller and null (after FDR correction) for 11–14 and 17–18 year age bins and additionally attenuated for age 5–6, 7–8, and 15–16 in adjusted models. Comparisons across severity, directionality, internalizing, and externalizing (Supplemental file 2, Section 3) confirmed that externalizing findings likely reflect a combination of MSDP effects on comorbid symptom severity and specificity of MSDP effects toward externalizing problems. For example, in the 1–2y age bin in the adjusted model, the standardized effects show similar magnitudes for comorbid symptoms (children exposed to MSDP were + 0.08 standard deviations higher on severity scores) and externalizing symptoms (children exposed to MSDP were + 0.10 standard deviations higher on externalizing scores). However, there were smaller effects for internalizing (0.05), which contributed to the severity score and were correlated with externalizing scores. Further, the association between MSDP and directionality (−0.07) indicates a specific effect on differentiation toward externalizing that is smaller in magnitude than the effect on the externalizing score (e.g., 0.10). This pattern was generally consistent across developmental age bins.

##### E-cigarette use

In the subset that included prenatal e-cigarette use, children were 10 years of age or younger. In adjusted models (supplemental file 1, Section 3), e-cigarette use was related to higher symptom severity only for the 7–8-year age bin (out of five total bins) but predicted lower symptom severity for the 1–2-year age bin. E-cigarette use was related to differentiation of problem type towards externalizing problems for the 3–4-year age bin, consistent with the MSDP findings, but was unrelated to directionality in the other four age bins. Due to wide confidence intervals, these data should be considered preliminary and interpreted with caution.

##### Other substances used during pregnancy and postnatal secondhand smoke exposure

There were sparse, unsystematic associations with other substances used during pregnancy after accounting for MSDP (Supplemental file 1, Section 4). Positive associations with symptom severity included prenatal opioid use at age 13–14 and cannabis at age 7–8, but there was a negative association between symptom severity and illicit drug use at age 9–10. Associations with differentiation toward internalizing symptoms included prenatal opioid use (ages 9–10, 13–14 and 17–18). Associations with differentiation toward externalizing symptoms included prenatal illicit drug use at age 9–10. These effects were not adjusted for FDR, and we believe they are likely chance findings.

Associations between postnatal secondhand smoke exposure and severity were generally small and positive though often null (Supplemental file 1, Section 5). Positive associations with symptom severity were found in childhood age bins and 13–14 years. Associations between postnatal secondhand smoke exposure and directionality were also small and mainly null: associations with differentiation toward externalizing problems were found at ages 5–6, 7–8, and 17–18 years.

## Discussion

We leveraged a large, sociodemographically diverse harmonized national dataset to characterize the associations of MSDP with child and adolescent symptom severity and symptom directionality (e.g., differentiation toward externalizing or internalizing domains) in two-year age bins spanning ages 1 to 18 years. Our use of symptom severity and directionality contributes to the literature by directly probing comorbidity vs. dimensional specificity of psychopathology symptoms. We generally found associations of MSDP with comorbid symptom severity in most age bins, after controlling for sociodemographic factors, family history of psychiatric conditions, other prenatal substance use, and postnatal secondhand smoke exposure. Our findings support associations between MSDP and increased comorbid symptom severity as well as support externalizing-specific associations with MSDP, with internalizing implicated primarily via comorbidity. This indicates that there are multiple pathways from MSDP to psychopathology symptoms in childhood and adolescence, supporting that multifinality is important to consider. At the same time, there are also likely domain-specific effects that strengthen the likelihood of exhibiting externalizing problems specifically when MSDP contributes to risk of psychopathology. Use of the ECHO Cohort also increases generalization of findings, given the socioeconomic, racial, ethnic, and regional diversity of the sample.

Our large sample allowed us to examine effects across two-year age bins, addressing the limitation in the literature that most studies to date have been unable to ascertain developmental-period specificity of associations. Findings were mostly consistent across age bins, with more evidence of fade-out than sleeper effects – although this may be somewhat confounded by the smaller sample sizes available for later age bins. As we increased adjustment (e.g., supplemental file 1, Section 1.5), findings for severity across early-to-mid childhood (< age 7) never attenuated; however, the effects of postnatal smoke exposure attenuated findings in preadolescence (age 7–8), and mid-to-late adolescence (< age 13) attenuated completely. Although the magnitude of associations decreased for early pubertal age bins (9–10, 11–12), MSDP still was associated with severity of problems after controlling for other prenatal substance use and postnatal smoke exposure. These findings corroborate the idea that early childhood and early puberty are two sensitive periods for the effects of MSDP on symptom severity or comorbid symptoms. However, it is important to highlight that the specific analyses with null effects—where effects attenuated—were nearly all from models with greatly reduced sample sizes. Further, for directionality, effects were generally fairly small, and instances where findings were vs. were not significant were generally for effects of the same magnitude across. Thus, overall, our findings suggest that there may not be sensitive periods for the effects of MSDP, pointing to the critical importance of prevention strategies to reduce MSDP prior to exposure and the need for interventions to reduce externalizing problems across childhood and adolescence in youth exposed to MDSP, rather than a critical window for prevention.

The large sample also allowed for testing of sex differences. We found very little evidence of sex differences—they were only present at age 13–14 years. The pattern of findings suggested boys had more severe comorbid problems than girls that also skewed toward a more dramatic preponderance of externalizing rather than internalizing directionality, though boys reported more externalizing and also more internalizing problems than girls at age 13–14 years. Because of the sparseness of findings, we urge caution in interpreting these findings as a specific developmental effect without replication.

Our use of statistical controls for other substances and postnatal secondhand smoke exposure strengthens confidence that the effects reported here are specific to prenatal tobacco as opposed to other substance use during pregnancy or postnatal exposures. Our findings differed somewhat from Ekblad and colleagues (2020), which found MSDP effects on differentiation toward externalizing problems but not comorbid symptom severity in a sibling comparison design of 8–15-year-old youth. We replicated similar externalizing-specific associations in some of the age bins but noted stronger effect sizes for symptom severity than for symptom directionality, in contrast to Ekblad and colleagues (2020). Given the robust genetic and environmental intergenerational transmission of comorbid symptom severity relative to symptom directionality (Marceau et al., 2024; Marceau & Neiderhiser, 2022; Marceau et al., 2022), the present findings for symptom severity may be inflated by unmeasured familial cofounders such as genetic risk and more stressful life experiences. Adding nuance, while some studies report that within-family analyses attenuated the associations between MSDP and child behavior (Estabrook et al., 2016; Knopik, Marceau, Bidwell, et al., 2016), Dolan et al. (2016) reported that the association between MDSP and externalizing behavior persisted in twin models. Nevertheless, due to our inability to test for within-family differences, we acknowledge that our findings may reflect unmeasured family-level confounding (D’Onofrio et al., 2010).

Finally, our ability to examine e-cigarette use contributes novel discovery to the field. Although we could not test all age bins due to limited data availability, we demonstrated some associations between e-cigarette use and childhood psychopathology outcomes. Although findings were less consistent than for cigarette smoking, this adds support to the notion that some MSDP effects may be in part driven by nicotine exposure and likely exacerbated or also driven by other components found in cigarette smoke (Knopik, 2009).

### Limitations

In the present study, the measurement of MSDP came from multiple sources but most often was self-reported retrospectively. While maternal self-report is considered valid (Knopik, Marceau, Palmer, et al., 2016), physiologically verified smoking during pregnancy exposure is the gold standard. Further, we were only able to capture whether MSDP occurred, as there was insufficient data to explicitly test timing, frequency and dose–response effects, which are important for understanding potential thresholds and sensitive periods that could be used to inform targeted prevention efforts. This limitation stemmed from having to harmonize exposure measures across cohorts which required reducing MDSP to a binary yes/no, thereby eliminating the possibility of making inferences related to dose, frequency, and timing for symptom severity and directionality of internalizing and externalizing symptoms. Despite the reduced specificity of the exposure assessment, we believe this study represents a valuable incremental step for examining MSDP at scale by leveraging a uniquely large and diverse sample, enhancing both generalizability and statistical power to detect small but potentially meaningful associations, particularly given that no other U.S. cohort of this size and representation currently allows for such an analysis. These findings lay the groundwork for future investigations using more detailed exposure data to better understand dose–response relationships and prenatal developmental timing effects. This tradeoff of utilizing a crude measurement of MSDP derived by harmonizing data from many cohorts to maximize the sample size is also true of our measures of other substance use during pregnancy and postnatal secondhand smoke measures.

Additionally, we were unable to show that the CBCL was measurement invariant across age bins or sex due to zero-variance items. This means that the underlying internalizing and externalizing scores, and also the severity and directionality scores on which they are built, may not have the same structure or meaning across age/sex bins, and thus direct comparisons between bins should not be emphasized or concluded—for example, sex differences or differences in findings across age bins could reflect measurement artifacts. Instead, we focused on the general pattern of results.

Finally, as noted above, this is not a genetically informed analysis, and we could not account for genetic confounding, although the inclusion of key covariates (e.g., postnatal smoking, family history of psychiatric disorder) does to some extent control for relevant familial influences. We can expect, based on prior literature, that the effects here are somewhat inflated by familial confounding and are not good causal estimates.

### Implications and conclusions

Utilizing a large, harmonized dataset from the well-established ECHO consortium, this study found that MSDP is associated with greater severity of comorbid symptoms and a specific risk for externalizing behaviors in children and adolescents for both boys and girls similarly across ages. These findings offer a new perspective on child neurobehavioral development and suggests that interventions targeting MSDP could meaningfully reduce comorbid and externalizing-specific neurobehavioral problems across development.

## Supplementary Material

Supplementary Materials

**Supplementary material.** The supplementary material for this article can be found at https://doi.org/10.1017/S095457942610128X.

## Figures and Tables

**Table 1.** Sample description

Description	Analytic sample			Full ECHO Cohort
	Split by MSDP exposure		Total	
	No	Yes		
	*N* = 14,674	*N* = 1,661	*N* = 16,335	*N* = 52,367
**MSDP Exposure**				
0, No			14,674 (90%)	37,497 (90%)
1, Yes			1,661 (10%)	4,225 (10%)
Missing			0 (0%)	10,645 (20%)
**Has CBCL data?**				
Yes	14,674 (100%)	1,661 (100%)	16,335 (100%)	20,120 (38%)
Missing				32,247 (62%)
**Mutually exclusive race category of the participant (combined Asian category)**				
1, White	8,223 (56%)	927 (56%)	9,150 (56%)	28,048 (54%)
2, Black	2,426 (17%)	372 (22%)	2,798 (17%)	8,327 (16%)
3, Asian	722 (5%)	6 (<1%)	728 (4%)	1,931 (4%)
4, Native Hawaiian or other Pacific Islander	37 (<1%)	9 (<1%)	46 (<1%)	160 (<1%)
5, American Indian or Alaska Native	346 (2%)	60 (4%)	406 (2%)	1,546 (3%)
6, Multiple Race	1548 (11%)	227 (14%)	1775 (11%)	5,040 (10%)
7, Other Race	796 (5%)	31 (2%)	827 (5%)	2,315 (4%)
Missing	576 (4%)	29 (2%)	605 (4%)	5,000 (10%)
**Ethnicity of the child participant, Hispanic or not**				
0, non-Hispanic	10,773 (74%)	1,410 (85%)	12,183 (75%)	36,921 (70%)
1, Hispanic	3,728 (25%)	234 (14%)	3,962 (24%)	13,079 (25%)
Missing	173 (1%)	17 (1%)	190 (1%)	2,367 (5%)
**Sex of the child at birth**				
1, Male	7,691 (52%)	839 (50%)	8,530 (52%)	27,088 (52%)
2, Female	6,982 (48%)	822 (50%)	7,804 (48%)	25,115 (48%)
3, ambiguous	0 (0%)	0 (0%)	0 (0%)	8 (<1%)
Missing	1 (<1%)	0 (0%)	1 (<1%)	156 (<1%)
**Age of the biological mother at the birth of the child participant**				
Mean (SD)	30.3 (5.9)	27.1 (5.8)	30.0 (5.9)	27.3 (11.1)
Median (Min – Max)	31.0 (14.0 – 58.0)	26.0 (14.0 – 47.0)	30.0 (14.0 – 58.0)	29.0 (13.0 – 58.0)
Missing	121 (<1%)	12 (<1%)	133 (<1%)	0 (0%)
**Highest level of maternal education**				
1, Less than high school	755 (5%)	245 (15%)	1,000 (6%)	3,920 (9%)
2, High school degree, GED or equivalent	1,595 (11%)	381 (23%)	1,976 (12%)	7,107 (16%)
3, Some college, no degree and above	12,139 (83%)	1,000 (60%)	13,139 (80%)	33,491 (75%)
Missing	185 (1%)	35 (2%)	220 (1%)	7,849 (15%)
**Child’s first degree relative (mother, father, sister, brother) ever had psychiatric disorder**				
0, No	4,490 (31%)	301 (18%)	4,791 (29%)	8,465 (16%)
1, Yes	5,028 (34%)	906 (55%)	5,934 (36%)	10,487 (20%)
Missing	5,156 (35%)	454 (27%)	5,610 (34%)	33,415 (64%)

*Note.* Most of the sample reduction was due to missing outcome data (61%). Due to rounding, some % values within don’t add up to 100.

**Table 2.** Prenatal substance use and postnatal secondhand smoke exposure by MSDP status

Variable	No MSDP *N* (% of non-missing)	Any MSDP *N* (% of non-missing)	Total *N* (% of non-missing)
**Prenatal alcohol Use**			
0, No	10,178 (84%)	1,023 (72%)	11,201 (83%)
1, Yes	1,941 (16%)	393 (28%)	2,334 (17%)
Missing	2,555	245	2,800
**Prenatal opioid use**			
0,No	11,294 (91%)	1,070 (82%)	12,364 (90%)
1, Yes	1,184 (9%)	229 (18%)	1,413 (10%)
Missing	2,196	362	2,558
**Prenatal Marijuana use**			
0, No	9,189 (96%)	814 (75%)	10,003 (94%)
1, Yes	371 (4%)	267 (25%)	638 (6%)
Missing	5,114	580	5,694
**Prenatal Other illicit drug use**			
0, No	7,673 (99%)	1,128 (92%)	8,801 (98%)
1, Yes	79 (1%)	96 (8%)	175 (2%)
Missing	6,922	437	7,359
**Postnatal secondhand smoke exposure**			
0, No	10,840 (84%)	466 (35%)	11,306 (79%)
1, Yes	2,117 (16%)	848 (65%)	2,965 (21%)
Missing	1,717	347	2,064

## Data Availability

Select de-identified data from the ECHO Program are available through NICHD’s Data and Specimen Hub (DASH). Data for this study were centrally collected following the execution of analysis-specific Data Use Agreements between each cohort and the ECHO Data Analysis Center (DAC) at Johns Hopkins University. Data cannot be shared publicly but researchers who meet the ECHO criteria for confidential data access can request access via contacting the ECHO Data Analysis Center: ECHO-DAC@rti.org. Information on study data not available on DASH, such as some Indigenous datasets, can be found on the ECHO study DASH webpage. All R code for reproducibility is available upon request of the second author.

## Footnotes

1 Standardized coefficients were calculated as the unstandardized coefficients*(standard deviation of predictor/standard deviation of outcome). Although the unstandardized estimates were computed from the pooled imputed data, we used the standard deviation of the raw data within each age bin to standardize the estimates from each age bin as a close approximation.

## Availability of code.

All R code for reproducibility is available upon request of the second author.

## Availability of methods/material.

Further details of methods/materials are available on the ECHO study website.
